# Flexible Foraging Response of Wintering Hooded Cranes (*Grus monacha*) to Food Availability in the Lakes of the Yangtze River Floodplain, China

**DOI:** 10.3390/ani10040568

**Published:** 2020-03-27

**Authors:** Zhenhua Wei, Meng Zheng, Lizhi Zhou, Wenbin Xu

**Affiliations:** 1 School of Resources and Environmental Engineering, Anhui University, 111 Jiulong Road, Hefei 230601, China; weizhenhua_ah@163.com (Z.W.); zhengmeng6688@126.com (M.Z.); 2 Anhui Province Key Laboratory of Wetland Ecosystem Protection and Restoration (Anhui University), 111 Jiulong Road, Hefei 230601, China; 3 The Forestry Department of Hunan Province, Changsha 410000, China; 4 Anhui Shengjin Lake National Nature Reserve, Dongzhi 247200, China; xuwenbin_sjlnnr@163.com

**Keywords:** *Grus monacha*, foraging behavior, foraging strategies, food availability, wintering ecology

## Abstract

**Simple Summary:**

With increasing human activity, bird habitats are being degraded and lost. In order to survive, birds have to adapt to the deteriorating environment. In this study, we surveyed the availability of food resources to Hooded Cranes in a degraded wetland. We found that cranes adopted flexible foraging strategies in response to the abundance and availability of different food resources in mosaic wetland landscapes at different periods during winter. The results also showed that the deeper the food was buried, the more time the cranes spent, and foraging frequency and foraging success rate were based on food abundance. It provided an evidence basis for the protection and management of waterbirds, especially Hooded Cranes.

**Abstract:**

Wetlands are disappearing or degrading at an unprecedented rate due to the increase in human encroachment and disturbance, eventually leading to habitat loss for waterbirds, which is the primary cause of the decline in the Hooded Crane (*Grus monacha*) population. The Hooded Cranes have to constantly adjust their foraging strategies to survive to cope with this situation. In order to study how cranes respond to food resources in mosaic habitat, we surveyed a total of 420 food quadrats and 736 behavioral samples from three habitats during three wintering periods in Shengjin Lake and Caizi Lake. We measured temporal and between-habitat differences in foraging time budget, foraging frequency, and foraging success rate. Akaike’s information criterion was selected between the models of food abundance and availability. The results indicated that the wintering cranes spent the majority of their time (66.55%) foraging and shifted their foraging behaviors based upon food abundance and availability in different habitats. Our analyses also indicated that cranes were willing to forage more food with poor sediment penetrability in sub-optimal habitats. Foraging time budget was based on the food depth, and the foraging frequency and foraging success rate were based on food abundance. Cranes adopted flexible foraging strategies in response to the alternative food resources in mosaic wetland habitats, as it could mitigate the negative impacts of habitat loss and facilitate survival.

## 1. Introduction

Foraging is a critical component of the annual life history of animals [1,2] and is a major determinant of survival [3,4], requiring the balancing of risk and reward [5,6]. Theoretically, it is accepted that habitat patches are complex, with food resources varying across different spatiotemporal scales [7,8]. Such heterogeneity in food resources has also been documented for real-world ecosystems [9]. The quality and quantity of food may also be inversely correlated [10], with areas containing less nutritious items tending to cover larger areas [11]. These constraints constitute an important ecological problem for species, with factors other than food also likely altering the foraging behavior of animals [12]. Given these constraints, animals have likely developed flexible foraging strategies to optimize the benefits of foraging to facilitate survival, and they are able to respond to the complex environment in a timely manner and adjust their foraging behavior to attempt to consume food resources that offer the greatest energy gain at the least cost [13,14,15]. Waterbirds, in particular, must display flexibility due to the constantly changing water levels of wetlands, as well as the management of agricultural lands (such as paddies) [13,16,17,18,19,20]. Migratory waterbirds display flexibility in foraging behaviors, e.g., dunlin have changed their foraging mode, as well as morphological and digestive strategies, in response to changes in environmental conditions [21,22]. This is because the fluctuating hydrology of aquatic systems alters the abundance and availability of the habitats that they occupy [23,24]. Consequently, waterbirds must exploit different food resources that offer high rates of energy gain [15,25]. However, increasing human encroachment of wetland habitats has largely disrupted natural hydrological processes, reducing suitable foraging habitats [26,27,28]. Waterbirds, especially larger ones (such as cranes and swans), have simultaneously begun to exploit human-modified habitats that offer food resources (e.g., agricultural habitats) [29,30]. Therefore, several recent studies have focused on the ability of waterbirds to use flexible foraging strategies when utilizing different landscapes to obtain sufficient food, particularly in areas subjected to habitat degradation and loss [14,31]. However, these adaptive responses might not be typical across species or geographic regions, with additional research being required.

The Hooded Crane (*Grus monacha* Temminck, 1835) is a typical large migratory bird species that is classified as vulnerable (VU) in the revised Red List of the International Union for Conservation of Nature (IUCN), with a global population of approximately 11,600 individuals [13]. Three sub-populations breed in the tundra and floodplains of eastern Siberia and northeast China [32,33]. After fledging, cranes migrate thousands of kilometers to wintering grounds in southern Korea, southern Japan, and eastern China [33,34]. Furthermore, the Yangtze River floodplain on the East Asian-Australian flyway is unique for its extensive ephemeral river-lake wetlands. The water levels of these wetlands are low from winter to early spring and are recharged by precipitation during the summer monsoons. Floodwater from these rains is laden with nutrients and sediment [35,36], creating one of the largest concentrations of shallow and seasonal flooded lake wetland networks globally [37]. The Shengjin and Caizi lakes represent two important wetlands in the middle and lower Yangtze River floodplain, providing critical foraging habitats for globally significant numbers of migratory waterbirds, supporting abundant and diverse food resources [36,38]. In parallel, the two lakes serve as primary wintering areas for the Hooded Cranes, with up to 600 individuals recorded [39].

However, in recent 15 years, these wetlands have been lost or degraded at an unprecedented rate because of increasing human encroachment and disturbance. These activities have reduced the availability of suitable foraging habitats for waterbirds [26,31,40]. The integrity of river-lake ecosystems is being threatened by increasing economic initiatives, particularly intensified aquaculture of fish [38], causing the widespread decline and collapse of submerged macrophytes and zoobenthos [41]. The degeneration and loss of habitat in traditionally suitable foraging patches have significantly threatened the Hooded Cranes in the two lake wetlands [34]. The loss of two aquatic plant species—*Vallisneria spinulosa* and *Potamogeton malaianus—*is of major concern because they represent primary food sources for cranes [13]. Consequently, cranes are being forced to use alternative food sources, particularly the spilled rice grains in paddy fields [26,39,42]. In practice, these alternative food items are vital for supporting the survival of cranes and buffering the negative impacts of primary habitat loss [14]. Thus, it is important to assess the quantity and quality of the food resources of cranes, as well as their ability to shift foraging behavioral patterns for future conservation efforts. To understand whether Hooded Cranes will respond positively to food resources in mosaic habitat, we (a) quantified differences in foraging behaviors, (b) focused on the impacts of the spatiotemporal dynamics in food resources on the foraging behaviors of cranes in alternative habitats during winter, and (c) evaluated the ability of cranes to optimize their foraging strategies by identifying trade-offs in food resource characteristics in different habitats in the lakes of the Yangtze River floodplain, Anhui, China. The results of this study are expected to provide insights on how to manage the wintering foraging habitats of Hooded Cranes in disturbed environments.

## 2. Materials and Methods 

### 2.1. Study Sites

**Figure 1:**
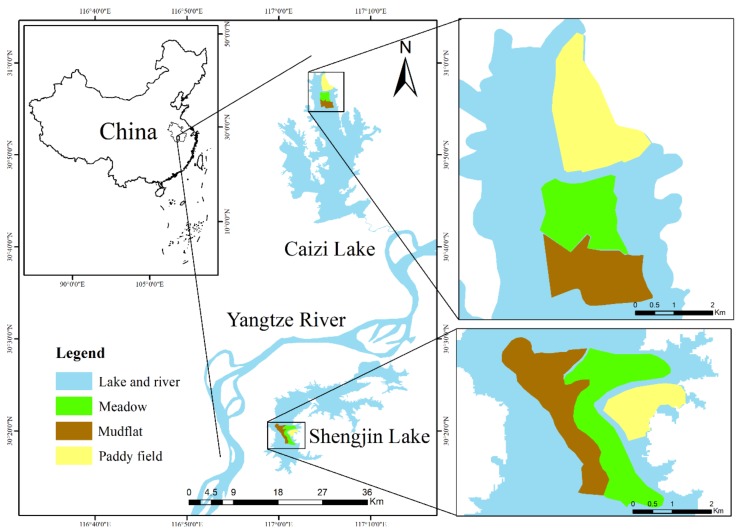
Location of the study area and sampling habitats in the middle and lower Yangtze River floodplain, Anhui Province, China.

The Shengjin (30°15′–30°45′ N, 116°55′–117°15′ E) and Caizi (30°43′–30°59′ N, 116°58′–117°11′ E) lakes are two typical river-connect shallow lakes that are located in the middle and lower Yangtze River floodplain, Anhui, China (Figure 1). Both lakes provide indispensable wintering and stopover habitat for migratory waterbirds on the East Asian-Australasian flyway [26,41,43], and Shengjin Lake is a Ramsar Site, a wetland of international importance. Hydrological fluctuations are largely influenced by the north subtropical humid monsoon climate, with a clear separation between dry and rainy seasons. The average annual temperature is 14–18 ℃, and the average annual rainfall is approximately 1000–1400 mm. Total variation in the extent of water at Shengjin Lake ranges from 140 km^2^ during the summer wet season to 75 km^2^ in the dry winter, resulting in the exposure of large areas of mudflats and small meadows of less than 20 km^2^, which provide suitable habitat for cranes [19,39,42,44]. Caizi Lake covers an area of 226 km^2^ in summer when water levels peak at 14.88 m and covers 114.97 km^2^ in winter when the water level drops to 10.63 m [41,45]. At low water levels, 60.57 km^2^ of mudflats and 44.61 km^2^ of meadows are exposed at Caizi Lake. Both lakes have small areas of paddy fields; namely, about 10 km^2^ at Shengjin Lake, and 20 km^2^ at Caizi Lake [46]. The three habitats (meadows, mudflats, and paddy fields) are the main foraging areas for Hooded Cranes and the sites where we delineated the food quadrats and observed foraging behaviors [13], about 500 individuals habitat here in recent years, with more than 200 individuals per lake. Until 2018, the main form of exploitation in these two lakes was the polyculture of fish and crabs, which has led to the destruction of submerged vegetation, such as *Vallisneria spinulosa* and *Potamogeton malaianus* [41].

### 2.2. Food Characteristics

From November 2012 to April 2013, we delineated 420 quadrats in total (10 m × 10 m) (Shengjin Lake and Caizi Lake each account half) (Table 1). In the center of each quadrat and at its four corners, food abundance and accessibility were recorded in smaller quadrats (0.5 m × 0.5 m). Plant tubers, mollusks, and rice grains were collected within 15 cm of the subsurface (water depth was not included), which is the typical foraging depth limit for crane species. The primary food sources of cranes in mudflats include *Anodonta woodiana* and the tubers of submerged vegetation, such as *Vallisneria spinulosa* and *Potamogeton malaianus*. The roots of *Potentilla supina*, *Ranunculus polii,* and *Polygonum criopolitanum* were collected from meadows, while rice grains (*Oryza sativa*) were collected from paddy fields.

All collected food items were washed and dried at 65 °C in an oven (YHG-9050A, Derip, Suzhou, China) for more than 72 h to determine the dry weight (g). We denoted food abundance (g/m^2^) as the ratio of the dry weight of food resources in a quadrat to the total quadrat area (0.25 m^2^). We also measured the depth of food (cm) and penetrability of the surrounding sediment as a function of food accessibility, which affected the cost of bill insertion and the ability to detect and capture food [21]. When the food is shallow and surrounded by less penetrable sediment, the accessibility of food is high for cranes. We surveyed the depth of each food item using a straightedge (Deli, Beijing, China). We quantified sediment penetrability (N/cm^2^) by vertically inserting a stratometer (TYD-2, Alisun, Hangzhou, China) in the sediment from the surface and recording the peak value. 

### 2.3. Observations of Cranes Behavior

From November 2012 to April 2013, we divided winter into three distinct periods, based on the natural climate, hydrological fluctuations, and the migration patterns of Hooded Cranes; namely, early (November and December), middle (January and February), and late (March and April) period [39]. During each period, we walked around the study area once a week along the set route and observed the behavior of cranes from 7:00 to 17:00. A target was randomly selected with a spotting scope (ATS 80 HD 20-60×80, Swarovski, Absam, Austria). We observed the behavior of a given individual using focal sampling for 20 min consecutive observations until it flew away [14,47]. We recorded 736 behavior samples from three habitats during three periods (Table 1).

Foraging behavior was classified as any behavior, including attempts to capture food, handling and consumption of food, including searching for food items, stabbing, pecking, nibbling, tugging, thrashing, and bill-flicking. During observations, we verbally recorded each behavior in a voice-activated tape-recorder (ICD-UX544F; Sony, Japan) and later transcribed and calculated the percentage of time that focal cranes engaged in each behavior. If the target moved out of sight (e.g., moved behind vegetation or other cranes) during foraging, we stopped the observation. If a bird clearly altered its behavior due to observer presence (e.g., gave a distress call, demonstrated excessive vigilance), we suspended observations until the cranes resumed normal behavior. To avoid multiple observations of the same individual, we examined one flock at a time, and we only observed one crane in each isolated flock (distance was more than 50 m between flocks) on any given day. To reduce the influence of severe weather on the results of observations, we did not record observations during strong winds, thick fog, or heavy snow. 

### 2.4. Data Analysis

To quantify the foraging time budget, we divided the total time that an individual engaged in each behavior by the total observation duration of each sample. We calculated the foraging frequency as the number of foraging attempts per unit foraging time (attempts/min). We considered a foraging attempt to be successful if we observed that the cranes swallowed food. We calculated the foraging success rate as the percentage of food captures out of the total number of foraging attempts.

Multiple linear regression analysis with an identical link function was used to examine the effects of habitat, period, food abundance, depth, and sediment penetrability on each foraging response variable (foraging time budget, foraging frequency, and foraging success rate). Based on the principle of parsimony and scientific plausibility, we developed a priori candidate model, which, for each variable, contained various additive variable combinations. The null model contained only the intercept and had no interactions because there is no strong ecological explanation. The global model included all combinations of the explanatory variables (habitat, period, food abundance, depth, and sediment penetrability). 

We ranked candidate models using Akaike’s information criterion (AIC) corrected for small sample size. We calculated the Akaike weights (wi) by R (3.6.1) (R Foundation for Statistical Computing, Vienna, Austria), which indicated the relative likelihood of a model, given the data. To reduce model selection bias, we implemented multimodal inference using a confidence set of models. The models with ΔAICc < 2 were selected as candidate models [48,49], and the parameter estimates based on the weighted averages of the parameters in their best models were calculated. We also calculated the relative importance of explanatory variables in the best model by summing w_i_ in models where it appeared within a candidate model set. Finally, we calculated the weighted parameter estimates and unconditional standard errors for the explanatory variables in each analysis, based on w_i_ for all candidate models. We considered the predictor variables with a selection probability >0.9 to be strongly supported by models.

Multicollinearity among fixed variables was examined using variance inflation factors (VIF) of the candidate models, and all VIF (<5.25) indicated little evidence for multicollinearity among fixed variables. The food abundance, food depth, sediment penetrability, foraging time budget, foraging frequency, and foraging success rate data all displayed non-normal distributions and homoscedasticity, so we compared these data among the three habitats and periods using non-parametric tests, (i.e., Mann-Whitney U test and Kruskal–Wallis test). All statistical analyses were carried out in R (3.6.1).

## 3. Results

### 3.1. Food Characteristics

**Figure 2:**
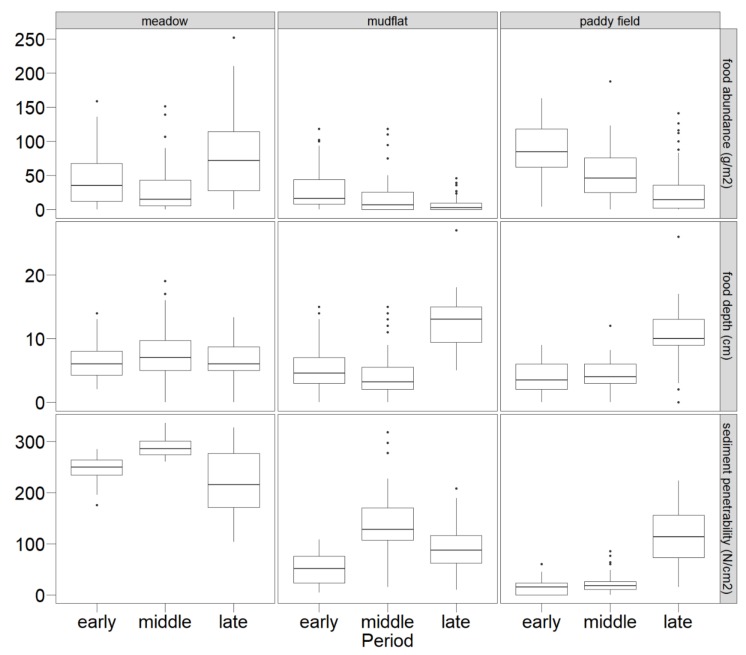
Food characteristics in foraging habitats during three wintering periods. The horizontal line between the box plot is the median, the boxes represent the interquartile range of data, the whiskers represent the range of data values for the lower 25% and upper 25%, and dots represent outliers.

In 420 quadrats, food abundance was 40.41 ± 44.78 g/m^2^ (Figure 2). There were significant differences in food abundance among the three foraging habitats (*χ*^2^ = 63.240, *p* < 0.001) and periods (*χ*^2^ = 24.705, *p* < 0.001). Food abundance in the mudflats and paddy fields declined, with minimum abundance occurring in meadows during the middle period. Food depth was 6.98 ± 4.59 cm. The depth of food in mudflats and paddy fields was relatively lower than that in the other habitats during the early and middle periods, and particularly during the late period. Sediment penetrability was 129.44 ± 98.94 N/cm^2^. Sediment penetrability in meadows was markedly greater than that in mudflats and paddy fields (*χ*^2^ = 254.168, *p* < 0.001), particularly during the middle period (*χ*^2^ = 33.783, *p* < 0.001).

### 3.2. Foraging Behaviors

**Figure 3:**
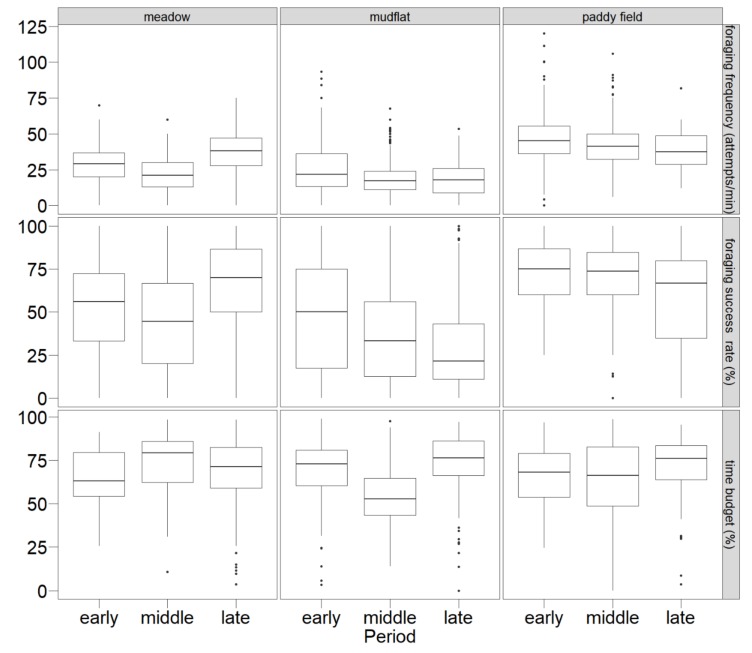
Fluctuations of the foraging time budget, foraging frequency, and foraging success rate in three habitats during three wintering periods. The horizontal line between the box plot is the median, the boxes represent the interquartile range of data, the whiskers represent the range of data values for the lower 25% and upper 25%, and dots represent outliers.

Across all habitats and periods, cranes spent 66.6 ± 20.4% of their time foraging at a frequency of 28.6 ± 16.9 attempts/min, with a success rate of 50.0 ± 30.4% (Figure 3). Foraging behaviors were significantly different in different periods (foraging time budge: *χ*^2^ = 26.586, *p* < 0.001; foraging frequency: *χ*^2^ = 231.882, *p* < 0.001; foraging success rate: *χ*^2^ = 84.869, *p* < 0.001) and habitats (foraging time budge: χ^2^ = 9.884, *p* = 0.007; foraging frequency: *χ*^2^ = 1118.958, *p* < 0.001; foraging success rate: *χ*^2^ = 646.075, *p* < 0.001). Cranes spent more time foraging in meadows (69.62 ± 18.55%) compared to mudflats (64.05 ± 20.68%) and paddy fields (65.79 ± 21.89%). Both foraging frequency and foraging success rates were greater in paddy fields (42.12 ± 15.61 attempts/min and 67.42 ± 24.22 %) compared to meadows (32.66 ± 14.37 attempts/min and 60.24 ± 27.85 %) and mudflats (20.31 ± 12.46 attempts /min and 35.10 ± 27.42%).

Although the foraging time in meadows did not decrease (*Z* = −1.939, *p* = 0.053), both foraging frequency and foraging success rates increased significantly (foraging frequency: *Z* = −11.400, *p* < 0.001; foraging success rate: *Z* = −8.178, *p* < 0.001). The decline in foraging frequency was continuous in mudflats and paddy fields, especially from middle to late period. Foraging success rate in mudflats declined rapidly from the early to middle period, while it declined more steadily in paddy fields (Figure 3). Compared with the early and middle period, there was a significant decrease from the middle to late period in paddy fields (foraging frequency: *Z* = −3.171, *p* = 0.002; foraging success rate: *Z* = −4.684, *p* < 0.001).

### 3.3. Behavioral Models of Foraging

The top-ranked model included food depth and sediment penetrability, which explained most of the variation in the foraging time budget of cranes (Table 2). A second model included only the sediment penetrability, and a third model included only food depth. Therefore, we model-averaged the parameter estimates in the three best models (Table 3). Both of the food characteristics were positive with cranes foraging time. The effect size was higher for food depth compared to sediment penetrability; however, neither parameter strongly supported the foraging time models.

Three candidate models were identified in the confidence sets for foraging success rate, and two candidate models were identified for foraging frequency, with △AIC_C_ values < 2. The top models for both foraging behaviors included habitat type and food abundance. Sediment penetrability and food depth also clearly affected the foraging success ratio of cranes (Table 2; Table 3). The model-averaged parameter estimates for the best models indicated that both food abundance and habitat type positively affected both foraging behaviors, whereas sediment penetrability negatively affected them. In comparison, only food depth negatively affected the foraging success rate. The effect size of food abundance among the food characteristics was the highest (Table 3).

## 4. Discussion and Conclusions

The food resources of the Hooded Cranes in the lakes of the middle and lower Yangtze River floodplain were not consistently available. Food was abundant and accessible during the early and middle periods in paddy fields, with the foraging attempts of cranes being more successful, resulting in less time being spent foraging than in the other habitats. The distance between the natural wetlands and paddy fields was relatively short. Cranes did not require much time, and hence little energy, to make frequent movements to adjacent habitats, devoting more time to foraging than other critical behaviors, such as resting [13,39]. The suitability of paddy fields as foraging habitat declined as grain became depleted, and the fields were plowed during the late period; however, paddy fields played a key role in meeting the energy requirements of cranes during the early and middle periods [42]. A similar decline in forage suitability was detected in mudflats, with the availability of mollusks rich in protein declining. Changes to meadows were strongly tied to seasonal phenology. During the early period, there was a good supply of roots and tubers of submerged and emergent vegetation, which did not wither entirely; however, cranes needed much more energy and time to acquire them compared to grains from paddy fields during the same period [14]. However, cranes spent more time foraging in meadows with an abundance of accessible roots and tubers from the middle to a late period, when temperature and precipitation began to rise. 

The foraging behavior of cranes also varied temporally. In particular, they must replenish their energy reserves that were expended during their migration and build up energy reserves for their return migration in the spring. After a long migration of more than two thousand kilometers, cranes must recover physically through rapid nutritional replenishment. During the early period, cranes spent more time and made more attempts to acquire food. Accordingly, cranes maximized their foraging success rate by foraging predominantly in paddy fields, where grains were abundant and accessible [42]. During the middle period, the foraging success rate dropped sharply because of the onset of cold, dry weather, and the subsequent hardening of the soil, which reduced the abundance and accessibility of food [26,41]. During this period, the cost of foraging increased, with cranes adaptively responding by decreasing their activity to regulate heat loss. This phenomenon was demonstrated by the minimal foraging rate that we observed. During the late period, due to roots and tubers being highly abundant in meadows, cranes shifted their dietary preferences to this habitat. During this period, cranes also increased the intensity of foraging activity because they needed to accumulate sufficient energy for migratory flight and to gain a size advantage over rivals on arrival at the breeding grounds [50]. 

Interestingly, the shifts in foraging habitat use were not only based on physiological drivers but the ability of cranes to respond to changes in habitat quality in relatively isolated habitat niches across the three periods. In meadows, cranes primarily shifted their foraging behavior in response to the penetrability of sediment and phenology of plants. Changes to the occupancy of mudflats by cranes appeared to be triggered by a period during which mollusks are dormant and the abundance of grains in paddy fields [26,42], apart from the high abundance of the meadow in the late period, the food abundance of the paddy field was the highest. Cranes always had access to multiple habitats, and we never observed them foraging in just one habitat. It is clear that the migratory birds, such as cranes, are highly mobile foragers that switch between different foraging habitats within landscapes in order to get the greatest energy gain, as the most profitable food resource changes over time [15]. Density-dependent declines in habitat suitability likely forced some individuals to occupy less suitable foraging habitats to reduce competition. In addition, animals that forage optimally should exercise “prudent predation” to balance energy intake and nutrient requirements [5,51]. Therefore, cranes adjusted their foraging behavior to selectively forage on each food type, depending on its characteristics (i.e., above certain density or size thresholds). The current study provided empirical evidence that appropriate adjustments in foraging behavior could extend to the ideal forager with respect to food and habitat choice [24].

Both the foraging frequency and foraging success rates directly depended on the abundance of food resources and the habitats supporting different types of food resources. Cranes preferentially fed on food resources that were abundant by moving among habitats; consequently, foraging behavior was not directly restricted by the same food characteristics. However, foraging behavior influenced the abundance and availability of food in different habitat patches [11,52], especially in severely degraded wetlands [14,26,53]. Previous studies have demonstrated that food abundance represents a primary factor influencing the time spent foraging by birds and mammals [4,11,21]. Recent studies have also shown that food abundance can be a key determinant of the rates of energy gain while foraging and that foragers may switch between adjacent habitats that differ in food abundance to maintain high rates of energy gain. We observed in our study that cranes were able to exploit a variety of habitats by moving among them. In addition, cranes modified their preferred foraging habitat, mainly utilizing habitats with abundant food resources in the composite ecological system. This result indicated that food abundance represented a key determinant of foraging time. Hence, foraging behaviors were based on food abundance [21].

Foraging behavior was also sensitive to variation in food depth and the penetrability of sediment [21,54]. Both of these parameters positively correlated with foraging time and negatively correlated with foraging frequency and foraging success rates. Cranes have a variety of foraging techniques; by using powerful bills flexibly, they pick up grains of rice on the ground, chisel through the shells of mollusks in mudflats, and dig roots and tubers from the depths of the soil [14,33,55]. Cranes sensitively exploit food resources from shallower soil depths and from sediment with lower hardness. It is more profitable for waders to reduce foraging time (and expend less energy) and invest more other behaviors [21,56], which increase the capture ratio, elevated nutrient intake rate, and avoided an energy-yield imbalance caused by attempting to forage on emergent aquatic vegetation that germinated too deep in hard sediment. Furthermore, cranes likely use effective strategies that protect their bills from wear and tear when excavating roots, pecking, and probing food resources.

Our findings highlighted flexible, individual-based foraging behaviors of cranes across spatiotemporal scales, with trade-offs occurring between food abundance and sediment penetrability. Our findings also showed that the quantity of food represented a key environmental constraint on foraging strategies. The conservation and restoration of foraging habitats, especially mudflats that are typically used by cranes, might facilitate the persistence of cranes at the population level. This study also highlighted the importance of paddy fields as alternative foraging habitat for cranes, which form a key component of their diet and might be of increasing importance with the continued loss of natural wetlands [26,57]. In conclusion, we suggested that the maintenance of fallow paddy fields should be strongly advocated, preserving them for as long as possible before the second plowing, or necessary rice grains should be provided as a key food supplement on the ground to help safeguard threatened Hooded Cranes populations.

## Figures and Tables

**Table 1:** The number of samples in different habitats during three wintering periods.

Lake	Period	Food Quadrats			Foraging Behavior Samples		
		Meadow	Mudflat	Paddy Field	Meadow	Mudflat	Paddy Field
Shengjin Lake	Early ^a^	25	25	20	11	18	35
	Middle ^b^	20	25	25	63	53	18
	Late ^c^	20	20	30	120	35	26
Caizi Lake	Early ^a^	20	25	20	14	45	29
	Middle ^b^	20	30	20	6	49	50
	Late ^c^	20	20	35	50	58	56
	Total	125	145	150	264	258	214

^a^ November and December, ^b^ January and February, ^c^ March and April.

**Table 2:** Candidate models for predicting Hooded Crane foraging behaviors in the mosaic wetland landscape. Models with △AICc >2 are not displayed (n=18) ^a^.

Model	AIC_C_^b^	△AIC_C_^C^	ML	*K*	W_i_	R^2^_adj_^d^
Foraging time budget						
Depth + penetrability	118.38	0	1.00	3	0.27	0.43
Penetrability	119.25	0.87	0.62	2	0.18	0.13
Depth	119.85	1.47	0.53	2	0.13	0.38
Null	124.11	15.73	0	1	0	
Foraging frequency						
Habitat + abundance	104.84	0	1.00	3	0.43	0.85
Habitat + abundance + penetrability	105.45	0.61	0.74	4	0.32	0.83
Null	130.47	25.63	0	1	0	
Foraging success rate						
Habitat + abundance + depth + penetrability	123.77	0	1.00	5	0.37	0.85
Habitat + abundance	124.29	0.52	0.76	3	0.28	0.82
Habitat + abundance + penetrability	125.54	1.77	0.41	4	0.15	0.81
Null	148.03	24.26	0	1	0	

AIC, Akaike’s information criterion; ML, Model Likelihood; *K*, The number of parameters; Wi, Akaike weight; R, Correlation coefficient; ^a^ All observations from a pool of cranes; ^b^ Akaike’s information criterion corrected for small sample size; ^c^ Difference between the AICc; ^d^ Adjust R-squared.

**Table 3:** Model-averaged parameter estimates and relative importance values for food characteristics affecting Hooded Crane foraging behaviors across wintering periods.

Response Variable	Explanatory Parameter	Estimate	SE	RI ^a^
Foraging time budget	Intercept	59.948	2.337	
	Depth	0.321	0.161	0.677
	Penetrability	0.027	0.014	0.741
Foraging frequency	Intercept	−3.370	6.858	
	abundance	0.495	0.049	1.000
	penetrability	−0.061	0.022	0.497
	Habitat	1.129	1.448	1.000
Foraging success rate	Intercept	3.361	10.426	
	Abundance	0.695	0.086	1.000
	Depth	−0.460	0.195	0.498
	Penetrability	−0.079	0.035	0.519
	Habitat	13.433	3.466	1.000

SE, Standard error; RI, Relative importance; ^a^ Relative importance value = sum of the Akaike weights for models including that variable.

## References

[1] Weimerskirch H., Ancel A., Caloin M., Zahariev A., Spagiari J., Kersten M., Chastel O. (2003). Foraging efficiency and adjustment of energy expenditure in a pelagic seabird provisioning its chick. J. Anim. Ecol..

[2] Heath J.P., Montevecchi W.A., Robertson G.J. (2008). Allocating foraging effort across multiple time scales: Behavioral responses to environmental conditions by Harlequin ducks wintering at Cape St. Mary’s, Newfoundland. Waterbirds.

[3] Rayner M.J., Hartill B.W., Hauber M.E., Phillips R.A. (2010). Central place foraging by breeding Cook’s petrel *Pterodroma cookii*: Foraging duration reflects range, diet and chick meal mass. Mar. Biol..

[4] Jakubas D., Wojczulanis-Jakubas K., Walkusz W. (2007). Response of dovekie to changes in food availability. Waterbirds.

[5] Macarthur R.H., Pianka E.R. (1966). On optimal use of a patchy environment. Am. Nat..

[6] Brown J.S., Kotler B.P. (2004). Hazardous duty pay and the foraging cost of predation. Ecol. Lett..

[7] Stephens D.W., Krebs J.R. (1986). Foraging Theory.

[8] Charnov E.L., Orians G.H., Hyatt K. (1976). Ecological implications of resource depression. Am. Nat..

[9] Wood K.A., Hilton G.M., Newth J.L., Rees E.C. (2019). Seasonal variation in energy gain explains patterns of resource use by avian herbivores in an agricultural landscape: Insights from a mechanistic model. Ecol. Model..

[10] Fryxell J.M. (1991). Forage quality and aggregation by large herbivores. Am. Nat..

[11] Hansen B.B., Aanes R., Herfindal I., Sæther B.-E., Henriksen S. (2009). Winter habitat–space use in a large arctic herbivore facing contrasting forage abundance. Polar Biol..

[12] Perry G., Pianka E.R. (1997). Animal foraging: Past, present and future. Trends Ecol. Evol..

[13] Wan W.J., Zhou L.Z., Song Y.W. (2016). Shifts in foraging behavior of wintering Hooded cranes (*Grus monacha*) in three different habitats at Shengjin Lake, China. Avian Res..

[14] Jia Y.F., Jiao S.W., Zhang Y.M., Zhou Y., Lei G.C., Liu G.H. (2013). Diet shift and its impact on foraging behavior of Siberian crane (*Grus Leucogeranus*) in Poyang Lake. PLoS ONE.

[15] Wood K.A., Stillman R.A., Wheeler D., Groves S., Hambly C., Speakman J.R., Daunt F., O’Hare M.T. (2013). Go with the flow: Water velocity regulates herbivore foraging decisions in river catchments. Oikos.

[16] DesGranges J.L., Ingram J., Drolet B., Morin J., Savage C., Borcard D. (2006). Modelling wetland bird response to water level changes in the Lake Ontario—St. Lawrence River hydrosystem. Environ. Monit. Assess..

[17] Holm T.E., Clausen P. (2006). Effects of water level management on autumn staging waterbird and macrophyte diversity in three Danish coastal lagoons. Biodivers. Conserv..

[18] Schmidt J.M., Sebastian P., Wilder S.M., Rypstra A.L. (2012). The nutritional content of prey affects the foraging of a generalist arthropod predator. PLoS ONE.

[19] Li C.L., Yang Y., Wang Z., Yang L., Zhou L.Z. (2018). The relationship between seasonal water level fluctuation and habitat availability for wintering waterbirds at Shengjin Lake, China. Bird Conserv. Int..

[20] Zhao Q.S., Wang X., Cao L., Fox A.D. (2018). Why Chinese wintering geese hesitate to exploit farmland. IBIS.

[21] Kuwae T., Miyoshi E., Sassa S., Watabe Y. (2010). Foraging mode shift in varying environmental conditions by dunlin *Calidris alpina*. Mar. Ecol. Prog. Ser..

[22] Zhang S.D., Ma Z.J., Choi C.Y., Peng H.B., Melville D.S., Zhao T.T., Bai Q.Q., Liu W.L., Chan Y.C., van Gils J.A. (2019). Morphological and digestive adjustments buffer performance: How staging shorebirds cope with severe food declines. Ecol. Evol..

[23] Naef-Daenzer L., Naef-Daenzer B., Nager R.G. (2000). Prey selection and foraging performance of breeding Great tits *Parus major* in relation to food availability. J. Avian Biol..

[24] Van Gils J.A., Spaans B., Dekinga A., Piersma T. (2006). Foraging in a tidally structured environment by Red knots (*Calidris canutus*): Ideal, but not free. Ecology.

[25] Nolet B.A., Bevan R.M., Klaassen M., Langevoord O., Van Der Heijden Y.G.J.T. (2002). Habitat switching by Bewick’s swans: Maximization of average long-term energy gain?. J. Anim. Ecol..

[26] Fox A.D., Cao L., Zhang Y., Barter M., Zhao M.J., Meng F.J., Wang S.L. (2011). Declines in the tuber-feeding waterbird guild at Shengjin Lake National Nature Reserve, China—A barometer of submerged macrophyte collapse. Aquat. Conserv. Mar. Freshw. Ecosyst..

[27] Baschuk M.S., Koper N., Wrubleski D.A., Goldsborough G. (2012). Effects of water depth, cover and food resources on habitat use of marsh birds and waterfowl in boreal wetlands of Manitoba, Canada. Waterbirds.

[28] Davidson N.C. (2014). How much wetland has the world lost? Long-term and recent trends in global wetland area. Mar. Freshw. Res..

[29] Wood K.A., Newth J.L., Brides K., Burdekin M., Harrison A.L., Heaven S., Kitchin C., Marshall L., Mitchell C., Ponting J. (2018). Are long-term trends in Bewick’s swan *Cygnus columbianus bewickii* numbers driven by changes in winter food resources?. Bird Conserv. Int..

[30] Beekman J., Koffijberg K., Wahl J., Kowallik C., Hall C., Devos K., Clausen P., Hornman M., Laubek B., Luigujõe L. (2019). Long-term population trends and shifts in distribution of Bewick’s swans *Cygnus columbianus bewickii* wintering in northwest Europe. Wildfowl.

[31] Peron G., Nicolai C.A., Koons D.N. (2012). Demographic response to perturbations: The role of compensatory density dependence in a North American duck under variable harvest regulations and changing habitat. J. Anim. Ecol..

[32] Ma Z.J., Li B., Jing K., Zhao B., Tang S.M., Chen J.K. (2003). Effects of tidewater on the feeding ecology of Hooded crane (*Grus monacha*) and conservation of their wintering habitats at Chongming Dongtan, China. Ecol. Res..

[33] Cai T.L., Huettmann F., Guo Y.M. (2014). Using stochastic gradient boosting to infer stopover habitat selection and distribution of Hooded cranes *Grus monacha* during spring migration in Lindian, northeast China. PLoS ONE.

[34] Harris J., Su L.Y., Higuchi H., Ueta M., Zhang Z.W., Zhang Y.Y., Ni X.J. (2000). Migratory stopover and wintering locations in eastern China used by White-naped cranes *Grus vipio* and Hooded cranes *G. monacha* as determined by satellite tracking. Forktail.

[35] Yang S.L., Zhao Q.Y., Belkin I.M. (2002). Temporal variation in the sediment load of the Yangtze River and the influences of human activities. J. Hydrol..

[36] Fang J.Y., Wang Z.H., Zhao S.Q., Li Y.K., Tang Z.Y., Yu D., Ni L.Y., Liu H.Z., Xie P., Da L.J. (2006). Biodiversity changes in the lakes of the central Yangtze. Front. Ecol. Environ..

[37] Yang X.D., Anderson N.J., Dong X.H., Shen J.I. (2008). Surface sediment diatom assemblages and epilimnetic total phosphorus in large, shallow lakes of the Yangtze floodplain: Their relationships and implications for assessing long-term eutrophication. Freshw. Biol..

[38] Cao L., Zhang Y., Barter M., Lei G. (2010). Anatidae in eastern China during the non-breeding season: Geographical distributions and protection status. Biol. Conserv..

[39] Zhou B., Zhou L.Z., Chen J.Y., Cheng Y.Q., Xu W.B. (2010). Diurnal time-activity budgets of wintering Hooded cranes (*Grus monacha*) in Shengjin Lake, China. Waterbirds.

[40] King S., Elphick C.S., Guadagnin D., Taft O., Amano T. (2010). Effects of landscape features on waterbird use of rice fields. Waterbirds.

[41] Chen J.Y., Zhou L.Z., Zhou B., Xu R.X., Zhu W.Z., Xu W.B. (2011). Seasonal dynamics of wintering waterbirds in two shallow lakes along Yangtze River in Anhui Province. Zool. Res..

[42] Zhao F.T., Zhou L.Z., Xu W.B. (2013). Habitat utilization and resource partitioning of wintering Hooded cranes and three goose species at Shengjin Lake. Chin. Birds.

[43] Dong Y.Q., Xiang X.J., Zhao G.H., Song Y.W., Zhou L.Z. (2019). Variations in gut bacterial communities of hooded crane (*Grus monacha*) over spatial-temporal scales. PeerJ.

[44] Yu C., Zhou L.Z., Mahtab N., Fan S.J., Song Y.W. (2019). The influence of food density, flock size, and disturbance on the functional response of Bewick’s swans (*Cygnus columbianus bewickii*) in wintering habitats. Animals.

[45] Li C.L., Li H.F., Zhang Y., Zha D.D., Zhao B.B., Yang S., Zhang B.W., de Boer W.F. (2019). Predicting hydrological impacts of the Yangtze-to-Huaihe Water Diversion Project on habitat availability for wintering waterbirds at Caizi Lake. J. Environ. Manag..

[46] Fang L., Dong B., Wang C., Yang F., Cui Y., Xu W., Peng L., Wang Y., Li H. (2020). Research on the influence of land use change to habitat of cranes in Shengjin Lake wetland. Environ. Sci. Pollut. Res..

[47] Altmann J. (1974). Observational study of behavior: Sampling methods. Behaviour.

[48] Maslo B., Burger J., Handel S.N. (2012). Modeling foraging behavior of piping plovers to evaluate habitat restoration success. J. Wildl. Manag..

[49] Burnham K.P., Anderson D.R. (2002). Model Selection and Multimodel Inference: A Practical Information-Theoretic Approach.

[50] Oppel S., Powell A.N., Butler M.G. (2011). King eider foraging effort during the pre-breeding period in Alaska. Condor.

[51] Slobodkin L.B. (1974). Prudent predation does not require group selection. Am. Nat..

[52] Gawlik D.E. (2002). The effects of prey availability on the numerical response of wading birds. Ecol. Monogr..

[53] Turner R.K., van den Bergh J.C.J.M., Söderqvist T., Barendregt A., van der Straaten J., Maltby E., van Ierland E.C. (2000). Ecological-economic analysis of wetlands: Scientific integration for management and policy. Ecol. Econ..

[54] Durell S.E.A.L.V.D. (2007). Individual feeding specialisation in shorebirds: Population consequences and conservation implications. Biol. Rev..

[55] Liu Q., Yang J.X., Yang X.J., Zhao J.L., Yu H.Z. (2010). Foraging habitats and utilization distributions of Black-necked cranes wintering at the Napahai Wetland, China. J. Field Ornithol..

[56] Lantz S.M., Gawlik D.E., Cook M.I. (2010). The effects of water depth and submerged aquatic vegetation on the selection of foraging habitat and foraging success of wading birds. Condor.

[57] Tourenq C., Bennetts R.E., Kowalski H., Vialet E., Lucchesi J.L., Kayser Y., Isenmann P. (2001). Are ricefields a good alternative to natural marshes for waterbird communities in the Camargue, southern France?. Biol. Conserv..

